# The efficacy and safety of telerehabilitation for patients following total knee arthroplasty: a overviews of systematic reviews

**DOI:** 10.1186/s12938-023-01158-z

**Published:** 2023-10-08

**Authors:** Danli Pang, Aiping Sun, Fei Wang, Jingyu Lu, Yaorui Guo, Wen Ding

**Affiliations:** 1 Department of Spinal Orthopedics, General Hospital of Ningxia Medical University, Yinchuan, 750000 China; 2 Ningxia Medical University, Yinchuan, 750000 China; 3 General Hospital of Ningxia Medical University, Yinchuan, 750000 China; 4 The First People’s Hospital of Yinchuan, Yinchuan, 750000 China; 5 Department of Nursing, General Hospital of Ningxia Medical University, No. 804 Shengli Street, Xingqing District, Yinchuan, 750000 Ningxia China

**Keywords:** Total knee arthroplasty, Telerehabilitation, Knee replacement, Overview, Systematic review

## Abstract

**Background:**

Studies evaluating the effectiveness and safety of telerehabilitation in patients undergoing total knee arthroplasty (TKA) have increased. However, the study quality and results differ, systematic reviews are limited. We aimed to synthesise systematic reviews and meta-analyses to assess the effects of telerehabilitation in patients post-TKA.

**Materials and methods:**

Systematic reviews and meta-analyses regarding the effectiveness and safety of TKA telerehabilitation were retrieved from eight databases from establishment to 18 December 2022. The Preferred Reporting Items for Systematic Reviews and Meta-Analyses (PRISMA), A Measurement Tool to Assess Systematic Reviews 2 (AMSTAR 2), Risk of Bias in Systematic Reviews (ROBIS) and GRADE system were used to evaluate results, methods, bias and evidence quality.

**Results:**

Thirteen systematic reviews and meta-analyses were analysed. The AMSTAR 2 showed low methodological quality in seven studies and very low quality in six. Among the key items, item 2 had been registered on website before systematic review in four reviews. Concerning item 4, did not provide a comprehensive search strategy in 4 reviews. For item 7, none of the reviews provided a list of reasons for excluding an article. For item 9, regarding whether appropriate tools were used to assess the risk of bias of each included study, one review was assessed as ‘partially yes’, one review only included RCTs, and the remainder were assessed as ‘yes’. For item 11, one review did not specify the statistical methods used, and three reviews did not conduct a meta-analysis. For item 13, four reviews considered the risk of bias when interpreting or discussing the study results. For item 15, seven reviews did not evaluate publication bias. The PRISMA scores of the 13 reviews ranged from 17.5 to 26.0. The PRISMA indicated that 69.2% had no protocol registration, 38.5% did not provide other materials and evidence certainty, 23.1% did not provide certainty assessment, 30.8% did not report study bias. According to the ROBIS scale, diferrent domains have diferrent risks in all the reviews.

**Conclusion:**

Telerehabilitation positively affects walking ability, knee extension and patient costs post-TKA surgery. Regarding the quality of life, patient satisfaction and the WOMAC, telerehabilitation had similar effects to conventional rehabilitation. Owing to the low quality of the studies, these conclusions should be interpreted cautiously, high-quality studies are needed in the future.

**Supplementary Information:**

The online version contains supplementary material available at 10.1186/s12938-023-01158-z.

## Introduction

Total knee arthroplasty (TKA) is the gold standard treatment of end-stage knee osteoarthritis or in cases wherein conservative treatment is ineffective [1]. Knee osteoarthritis is a degenerative knee disease with a prevalence of 85% in individuals over 65 years [2]. The number of TKA surgeries has increased globally, with more than 1,000,000 performed annually in the United States [3]. Early post-operative rehabilitation is critical to relieving pain, enhancing muscle strength, preventing complications such as deep vein thrombosis, joint stiffness and wound infection [4], promoting joint function recovery and improving patients’ independent living ability and quality of life.

With the development of rapid rehabilitation, hospitalisation times for patients undergoing TKA have shortened [5], with rehabilitation performed at home and during regular outpatient follow-ups. However, owing to geographical restrictions, implementing standard guidelines and supervision by healthcare professionals may be limited, and patients who have undergone TKA may lack functional exercise-related knowledge. Complications such as pain and wound swelling may not resolve over time [6], and dependence on rehabilitation is low. Studies have shown that 10–30% of patients with TKA had no significant improvement in joint function and poor recovery of physical function, and reoperation could likely result in a high economic burden on individuals and society [7]. Therefore, the post-operative rehabilitation needs of patients undergoing TKA are increasing; with the development of information technology, conventional rehabilitation faces challenges. Telerehabilitation refers to remote medical treatment through communication and information technologies such as the internet, smartphones, virtual reality, video phones and wearable electronic devices, providing patients with timely rehabilitation guidance, remote supervision and no travel distance [8]. Compared with conventional rehabilitation, remote rehabilitation saves time and cost, enables patients to obtain more convenient home rehabilitation services, enhances enthusiasm for rehabilitation, helps ensure continuous nursing care and improves the quality of life [9–11].

Systematic reviews of the effects of telerehabilitation in patients undergoing TKA have gradually increased in recent years. However, the quality of the studies, reported results and conclusions appear to differ, making clinical decision making challenging. Re-evaluation of systematic reviews involves comparing, summarising and synthesising these systematic reviews and meta-analyses regarding the same or similar interventions, which could provide comprehensive and reliable evidence for clinical decision makers [12]. Therefore, this study aims to evaluate systematic reviews and meta-analyses of the effectiveness and safety of telerehabilitation in patients undergoing TKA regarding method, report and evidence quality and risk of bias to provide a reference for facilitating clinical decision making.

## Results

### Literature screening process and results

A total of 1019 studies were preliminarily retrieved, of which 853 remained after removing duplicates using EndNote X9. A total of 821 studies were excluded after reading the titles and abstracts; 32 were read in full text, of which 19 were excluded, and 13 were finally included. The literature screening process is shown in, and the reasons for exclusion are shown in Additional file 2: Appendix Table 2.

#### Basic features of the included studies

Of the 13 systematic reviews, one was published in Chinese and 12 were published in English between 2017 and 2022. The number of original studies ranged from 4 to 28, and the sample size ranged between 230 and 6418 participants. All systematic reviews included randomised controlled trials (RCTs); some included prospective, observational and case–control studies. The Cochrane bias risk assessment tool was used in five studies, the Physiotherapy Evidence Database (PEDro) scale was used in four, the Joanna Briggs Institute RCT assessment tool was used in two, the Jadad scale was used in one, and the quality assessment tool was not specified in one. The intervention groups underwent remote rehabilitation, including videoconferencing and virtual reality rehabilitation, whereas the control groups underwent conventional rehabilitation and routine nursing (Table 1).

**Table 1:** Basic characteristics of the included studies (n = 13)

First author	Year	Country	Number of original studies (sample size)	Research type	Intervening measure	Comparison intervention	Outcome	Quality evaluation tool
McKeon, et al. [18]	2021	America	28 (6418)	RCT	Tele-rehabilitation	Face-to-face rehabilitation	WOMAC, KOOS, TUGT, satisfaction,cost	Not specified
McDonnell, et al. [19]	2022	Ireland	11 (1054)	RCT	Video-conferencing	Face-to-face rehabilitation	Satisfaction,VAS, WOMAC,TUGT	Cochrane bias risk assessment tool
Jansson, et al. [20]	2022	Finland	9 (1266)	RCT	Tele-rehabilitation	Face-to-face outpatient treatment	Joint flexion and extension, TUGT, VAS, life quality,WOMAC, KOOS	JBI randomized controlled trial evaluation tool
Jiang, et al. [26]	2018	China	4 (422)	RCT	Tele-rehabilitation	Face-to-face rehabilitation	VAS, WOMAC, active buckling and extension	Jadad scale
Gazendam, et al. [27]	2022	Canada	9 (835)	RCT	Virtual reality rehabilitation	Face-to-face rehabilitation	VAS, WOMAC, KOOS, cost	Cochrane risk assessment tool
Wang, et al. [30]	2021	Australia	11 (1020)	RCT	Internet rehabilitation	Face-to-face outpatient rehabilitation, home visits or routine care	Pain, knee flexion and extension, quality of life, satisfaction	JBI randomized controlled trial evaluation tool
Jing, et al. [21]	2019	China	6 (601)	RCT	Tele-rehabilitation	Routine outpatient physical therapy or home visit	WOMAC, joint flexion and extension, VAS, KOOS, TUGT	Cochrane Manual 5.1.0
Wang, et al. [23]	2019	Australia	21 (2188)	RCT	Tele-rehabilitation	Routine care	VAS, TUGT, WOMAC, life quality	PEDro scale
Tsang, et al. [24]	2022	China	11 (1825)	RCT	Tele-rehabilitation	Standard rehabilitation therapy	VAS, WOMAC, KOOS, cost, adverse event	PEDro scale
Peng, et al. [28]	2021	China	8 (805)	RCT	Virtual reality rehabilitation	Standard rehabilitation therapy	VAS, WOMAC, TUGT, life quality	PEDro scale
Blasco, et al. [29]	2021	Spain	6 (312)	RCT, case–control study	Virtual reality rehabilitation	Standard rehabilitation therapy	Pain, patient satisfaction	PEDro scale
Shukla, et al. [25]	2017	India	6 (408)	RCT, observational study	Tele-rehabilitation	Conventional rehabilitation	Active knee flexion and extension, VAS, life quality	Cochrane bias risk tool
Yoo, et al. [22]	2022	Korea	9 (230)	RCT, prospective study	Robot-assisted rehabilitation	Usual care	VAS	Cochrane bias risk tool, NOS scale

*JBI* Joanna Briggs Institute model of evidence-based healthcare, *KOOS* knee injury and osteoarthritis outcome score, *RCT* randomized controlled trial, *TKA* total knee arthroplasty, *TUGT* timed up and go test, *VAS* pain visual analogue score, *WOMAC* Western Ontario and McMaster Universities osteoarthritis index

### Results of methodological quality evaluation

Among the 13 systematic reviews included, seven [20, 22, 26–30] had low methodological quality, and six [18, 19, 21, 23–25] had very low methodological quality. Among the key items, item 2 in four reviews [22, 23, 28, 29] had been registered on a website before commencing the systematic review. Concerning item 4, four reviews [18, 19, 21, 26] did not provide a comprehensive search strategy. For item 7, none of the reviews [18–30] provided a list of reasons for excluding an article. For item 9, regarding whether appropriate tools were used to assess the risk of bias of each included study, one review [18] was assessed as ‘partially yes’, one review [25] only included RCTs, and the remainder were assessed as ‘yes’. For item 11, one review [25] did not specify the statistical methods used, and three reviews [18, 20, 29] did not conduct a meta-analysis. For item 13, four reviews [19, 22, 26, 28] considered the risk of bias when interpreting or discussing the study results. For item 15, seven reviews [19, 22–24, 26, 28, 30] did not evaluate publication bias. Among the noncritical weaknesses and in relation to item 10, none of the reviews [18–30] described the funding source (Table 2).

**Table 2:** Quality evaluation results of AMSTAR 2 included literature (n = 13)

Review	1	2	3	4	5	6	7	8	9	10	11	12	13	14	15	16	Grade
McKeon, et al. [18]	Y	N	Y	N	Y	Y	P	Y	P	N	NM	NM	N	Y	NM	Y	CL
McDonnell, et al. [19]	Y	N	Y	P	Y	Y	P	P	Y	N	Y	Y	Y	Y	N	Y	CL
Jansson et al. [20]	Y	P	Y	Y	Y	Y	P	Y	Y	N	NM	NM	N	N	NM	Y	Low
Liu, et al. [21]	Y	N	Y	P	Y	Y	P	Y	Y	N	Y	N	N	Y	Y	Y	CL
Yoo, et al. [22]	Y	Y	Y	Y	Y	Y	P	Y	Y	N	Y	Y	Y	N	N	Y	Low
Wang, et al. [23]	Y	Y	Y	Y	Y	Y	P	Y	Y	N	Y	Y	N	Y	N	Y	CL
Tsang, et al. [24]	Y	P	Y	Y	Y	Y	P	Y	Y	N	Y	N	N	Y	N	Y	CL
Shukla, et al. [25]	N	N	Y	Y	N	Y	P	P	RCT	N	N	Y	N	Y	Y	Y	CL
Jiang, et al. [26]	Y	P	Y	P	N	N	P	Y	Y	N	Y	N	Y	Y	N	Y	Low
Gazendam, et al. [27]	Y	P	Y	Y	Y	Y	P	P	Y	N	Y	Y	N	N	Y	Y	Low
Peng, et al. [28]	Y	Y	Y	Y	Y	Y	P	Y	Y	N	Y	N	Y	Y	N	Y	Low
Blasco, et al. [29]	Y	Y	N	Y	N	Y	P	P	Y	N	NM	NM	N	N	NM	Y	Low
Wang, et al. [30]	Y	P	Y	P	Y	Y	P	Y	Y	N	Y	Y	Y	Y	N	Y	Low

*N* no, *Y* yes, *P* partly yes, *NM* no meta-analysis, *RCT* randomized controlled trial, *CL* Critically low

### Literature report quality evaluation results

The PRISMA scores of the 13 systematic reviews ranged from 17.5 to 26.0. No systematic review reported serious defects, three reported some defects [18, 26, 30], and 10 reports were relatively complete [19–25, 27–29]. The title, abstract, introduction, source of information, study selection, study characteristics, study risk of bias and discussion have been fully reported in all reviews [18–30]. Nine reviews [18–21, 24–27, 30] did not register protocols; five [18–21, 26] did not provide the availability of data, code and other materials; three [18, 24, 30] did not provide certainty assessment, five [18, 22, 28–30] did not report the certainty of evidence; and four [18, 20, 24, 28] did not report study bias (Table 3).

**Table 3:** PRISMA evaluation results of the included literature (n = 13)

Section/topic	Items	McKeon, et al. [18]	McDonnell, et al. [19]	Jansson, et al. [20]	Liu, et al. [21]	Yoo, et al. [22]	Wang, et al. [23]	Tsang, et al. [24]	Shukla, et al. [25]	Jiang, et al.[26]	Gazendam, et al. [27]	Peng, et al. [28]	Blasco, et al. [29]	Wang, et al. [30]	Total points
Title	T1	Y	Y	Y	Y	Y	Y	Y	Y	Y	Y	Y	Y	Y	13.0
Abstract	T2	Y	Y	Y	Y	Y	Y	Y	Y	Y	Y	Y	Y	Y	13.0
Introduction	T3	Y	Y	Y	Y	Y	Y	Y	Y	Y	Y	Y	Y	Y	13.0
	T4	Y	Y	Y	Y	Y	Y	Y	Y	Y	Y	Y	Y	Y	13.0
Method	T5	Y	Y	Y	Y	Y	P	Y	P	P	Y	Y	Y	Y	11.5
	T6	Y	Y	Y	Y	Y	Y	Y	Y	Y	Y	Y	Y	Y	13.0
	T7	P	P	Y	P	Y	Y	Y	Y	P	Y	Y	Y	P	10.5
	T8	N	Y	Y	Y	Y	Y	Y	N	Y	Y	Y	P	Y	10.5
	T9	Y	Y	Y	Y	Y	Y	Y	P	P	Y	Y	Y	P	11.5
	T10	Y	Y	Y	Y	Y	Y	Y	P	Y	Y	Y	P	Y	12.0
	T11	Y	Y	Y	P	Y	Y	Y	Y	P	P	Y	Y	Y	11.5
	T12	N	Y	Y	Y	Y	Y	Y	Y	Y	Y	Y	Y	Y	12.0
	T13	N	Y	P	Y	P	Y	Y	N	Y	Y	Y	Y	P	9.5
	T14	N	Y	N	Y	P	Y	N	Y	Y	P	N	P	P	7.0
	T15	N	P	P	P	P	P	N	P	P	P	P	P	N	5.0
Result	T16	Y	Y	Y	Y	Y	Y	Y	Y	Y	Y	Y	Y	Y	13.0
	T17	Y	Y	Y	Y	Y	Y	Y	Y	Y	Y	Y	Y	Y	13.0
	T18	Y	Y	Y	Y	Y	Y	Y	Y	Y	Y	Y	Y	Y	13.0
	T19	Y	Y	Y	Y	Y	Y	Y	Y	Y	Y	Y	N	P	11.5
	T20	P	Y	Y	Y	Y	Y	P	Y	Y	P	Y	Y	P	11.0
	T21	P	Y	N	Y	Y	Y	N	Y	N	Y	Y	Y	Y	9.5
	T22	N	P	P	P	N	Y	P	P	P	Y	N	N	N	5.0

*N* no, *P* partly yes, *Y* yes, *T1* title, *T2* abstract, *T3* Rationale, *T4* objectives, *T5 *Eligibility criteria, *T6* information sources, *T7* search strategy, *T8* Selection process, *T9* Data collection process, *T10* data items; *T11 *Study risk of bias assessment, *T12* Effect measures; *T13* Synthesis methods, *T14* Reporting bias assessment, *T15* Certainty assessment, *T16* study selection, *T17* study characteristics, *T18* risk of bias in studies, *T19* results of individual studies, *T20* Results of syntheses, *T21* Reporting biases, *T22* Certainty of evidence, *T23* discussion, *T24* registration and protocol, *T25* support, *T26* Competing interests, *T27* Availability of data, code and other materials

### Biased risk assessment results

According to the ROBIS scale domain 1, the inclusion criteria, all the reviews (100%) were low risk. Regarding domain 2, research identification and selection, all reviews (100%) were high risk. In terms of domain 3, data extraction and quality assessment, two reviews (15.4%) were high risk. Regarding domain 4, data synthesis and results presentation, 10 reviews (76.9%) were high risk; in stage 3, all reviews (100%) had a high risk of bias, as shown in Table 4.

**Table 4:** Bias risk assessment results of included literature (n = 13)

Review	Phase 2				Phase 3
	Research inclusion criteria	Research identification and selection	Data extraction and quality evaluation	Data synthesis and results presentation	Risk of bias
McKeon, et al. [18]	√	×	×	?	×
McDonnell, et al. [19]	√	×	√	×	×
Jansson, et al. [20]	√	×	√	×	×
Liu, et al. [21]	√	×	?	×	×
Yoo, et al. [22]	√	×	√	?	×
Wang, et al. [23]	√	×	√	×	×
Tsang, et al. [24]	√	×	√	×	×
Shukla, et al. [25]	√	×	?	×	×
Jiang, et al. [26]	√	×	×	√	×
Gazendam, et al. [27]	√	×	√	×	×
Peng, et al. [28]	√	×	√	×	×
Blasco, et al. [29]	√	×	√	×	×
Wang, et al. [30]	√	×	√	×	×

*√,* low risk; × , high risk; ?, not sure

### Evidence quality evaluation results

The evidence quality evaluation results showed that among the 59 outcome indicators in the 13 reviews, there was no high-quality evidence, and there were 14 medium-quality and 33 low-quality indicators. The main reason for downgrade was the limitations of the original study, followed by publication bias and inconsistency, as shown in Table 5.

**Table 5:** Summary of evidence and grade evidence quality evaluation results of included studies (n = 13)

Review	Outcomes	Studies (participants)	Heterogeneity	Relative effect (95% CI)	P value	Grade level
McKeon, et al. [18]	WOMAC	12 (1325)	NR	NR	NR	Low
	KOOS	12 (363)	NR	NR	P < 0.001	Low
	TUGT	5 (499)	NR	NR	P = 0.30	Low
	Patient satisfaction	7 (1832)	NR	NR	NR	Low
	Cost	4 (1275)	NR	NR	NR	Low
	Pain	3 (281)	NR	NR	P > 0.05	Low
McDonnell et al. [19]	Patient satisfaction	4 (287)	0%	RR = 0.98(0.90–1.07)	P = 0.52	Moderate
	Pain	4 (294)	25%	SMD = − 0.33(− 0.61, − 0.65)	P = 0.02	Moderate
	WOMAC	3 (294)	0%	SMD = 0.12(− 0.11, 0.35)	P = 0.30	Low
	TUGT	6 (397)	73%	SMD = 0.19(− 0.25, 0.63)	P = 0.40	CL
Jansson, et al. [20]	Joint flexion	3 (255)	NR	NR	P > 0.05	low
	Joint extension	4 (315)	NR	SMD = − 0.06 (− 0.55–0.43)	P > 0.05	Low
	TUGT	3 (267)	NR	NR	NR	Low
	WOMAC	3 (449)	NR	NR	P < 0.05	Low
	KOOS	3 (427)	NR	NR	P > 0.05	Low
	Pain	5 (477)	NR	NR	P > 0.05	Low
	Life quality	1 (400)	NR	NR	P < 0.05	Low
	Adverse event	1 (225)	NR	NR	P < 0.05	Low
	Cost	4 (467)	NR	MD = − 263 (382–143)	P < 0.001	Low
Liu, et al. [21]	WOMAC	4 (532)	90%	MD = − 0.32(− 2.30 ~ 1.65)	P = 0.75	CL
	Joint flexion	4 (532)	86%	MD = 0.68 (− 2.28 ~ 3.63)	P = 0.65	CL
	Pain	3 (236)	0%	MD = − 0.43 (− 0.85 ~ − 0.01)	P = 0.04	Moderate
	KOOS	2 (234)	0%	MD = − 1.10 (− 1.63 ~ − 0.57)	P < 0.001	CL
	TUGT	3 (248)	84%	MD = − 5.17 (− 9.79 ~ − 0.55)	P = 0.03	Low
Liu, et al. [21]	Joint extension	3 (512)	0%	MD = 0.30 (0.20 ~ 0.40)	P < 0.001	Moderate
Yoo, et al. [22]	Pain	2 (42)	5%	SMD = 1.05 (0.39–1.71)	P = 0.30	Low
Wang, et al. [23]	Pain	3 (409)	0%	MD = − 0.19(− 0.36, − 0.03)	P = 0.02	Moderate
	TUGT	2 (207)	60%	MD = − 073(-11.18,-2.88)	P = 0.0009	CL
	WOMAC	4 (746)	15%	MD: − 0.09 (− 0.22, 0.04)	P = 0.18	Low
	Life quality	6 (681)	NR	NR	P = 0.05	Low
Tsang, et al. [24]	Joint extension	4 (699)	0%	SMD = − 0.19 (− 0.34 ~ − 0.04)	P = 0.01	Low
	Joint flexion	5 (886)	45%	SMD = 0.12(− 0.07–0.30)	P = 0.12	Low
	Pain	7 (787)	74%	SMD = -0.15(− 0.47–0.16	P = 0.34	CL
	WOMAC	8 (1219)	83%	SMD = 0.23(− 0.44 ~ 0.91)	P = 0.50	CL
	Cost	2 (316)	NR	NR	p < 0.001	Low
	KOOS	4 (804)	NR	NR	NR	Low
	Adverse event	4 (1165)	NR	NR	P = 0.007	Low
Shukla, et al. [25]	Joint extension	3 (248)	0%	MD = − 0.52 (− 1.39, 0.35)	P = 0.24	Low
	Joint flexion	3 (248)	0%	MD = − 0.52 (− 1.39, 0.35)	P = 0.24	CL
	Pain	2 (302)	0%	MD = − 0.02 (− 0.46–0.41)	P = 0.91	CL
	Life quality	1 (160)	NR	MD = 0.39 (0.46–7.34)	P = 0.03	CL
Jiang, et al. [26]	Pain	2 (161)	0%	MD = 0.52 (− 0.20–1.24)	P = 0.16	Low
	WOMAC	2 (207)	0%	MD = 1.13 (0.23–2.02)	P = 0.014	Low
	Joint flexion	2 (198)	0%	MD = 2.40 (− 0.34–5.15)	P = 0.09	Low
	Joint extension	3 (396)	0%	MD = 0.30 (0.20–0.40)	P < 0.00001	Moderate
Gazendam, et al. [27]	Pain	3 (282)	84%	MD = − 3.30 (− 8.03,1.43)	P = 0.17	CL
	WOMAC KOOS	4 (457)	73%	MD = − 3.32 (− 5.20 ~ − 1.45)	P = 0.08	Low
	Cost	1 (306)	NR	NR	p < 0.001	CL
Peng L, et al. [28]	Pain	3 (360)	91%	SMD = − 0.44 (− 0.79 ~ − 0.08)	P = 0.02	Moderate
	WOMAC	4 (257)	0%	SMD = − 0.71 (− 1.03 ~ − 0.40)	p < 0.01	Moderate
	TUGT	2 (163)	77.9%	SMD = − 0.34 (− 1.31–0.63)	P = 0.03	Low
	Life quality	2 (161)	NR	NR	NR	Low
Blasco J, et al. [29]	Pain	2 (192)	NR	NR	NR	Moderate
	Patient satisfaction	3 (107)	NR	NR	NR	Low
Wang, et al. [30]	Pain	6 (794)	48%	SMD = − 0.11(− 0.32–0.10)	P = 0.30	Moderate
	Joint flexion	4 (446)	0%	MD = 0.65(− 1.18, 2.48)	P = 0.48	Moderate
	Joint extension	4 (446)	0%	MD = − 0.38(-1.16, 0.40)	P = 0.34	Moderate
	Life quality	5 (652)	7%	SMD = − 0.09(− 0.26, 0.07)	P = 0.26	Moderate
	Patient satisfaction	3 (512)	0%	SMD = 0.04(− 0.21–0.14)	P = 0.67	Moderate

*SMD* standardized mean difference, *MD* mean difference, *RR* relative risk, *NR* not report, *CL* Critical low

*JBI* Joanna Briggs Institute model of evidence-based healthcare, *KOOS* knee injury and osteoarthritis outcome score, *RCT* randomized controlled trial, *TKA* total knee arthroplasty, *TUGT* timed up and go test, *VAS* pain visual analogue score, *WOMAC* Western Ontario and McMaster Universities osteoarthritis index

### Evaluation results of main outcome indicators

#### Pain

Thirteen reviews [18–30] evaluated pain. Two reviews [19, 21] showed statistically significant pain improvement in telerehabilitation groups compared with control groups, whereas 10 reviews [18, 20, 22–27, 29, 30] showed that telerehabilitation had no statistically significant effect on pain in patients who underwent TKA. Subgroup analysis in one review [28] showed that virtual reality rehabilitation could improve pain within 1 month after TKA surgery (SMD = − 0.44, 95% CI − 0.79–0.08, *P* = 0.02) but did not improve pain 2–3 months after surgery (SMD = − 0.35; 95% CI − 1.02–0.32, *P* = 0.31).

#### Knee function

Regarding the WOMAC indices, nine reviews [18–21, 23, 24, 26–28] evaluated the effect of telerehabilitation on WOMAC scores, six reviews [18–21, 23, 24] showed that the effects of telerehabilitation in terms of WOMAC scores were similar to those for standard care and three reviews [26–28] showed statistically significant effects of telerehabilitation in terms of WOMAC scores.

In terms of KOOS, five reviews [18, 20, 21, 24, 27] reported the effects of telerehabilitation on KOOS. Three reviews [18, 21, 27] showed that the telerehabilitation groups had improved KOOS compared with the control groups. In contrast, two other reviews [20, 24] showed no significant difference in KOOS between the telerehabilitation and control groups.

Regarding knee flexion and extension, six reviews [20, 21, 24–26, 30] evaluated knee flexion and extension and joint flexion, and the results showed that the effect of telerehabilitation on knee flexion was not statistically significant. In terms of joint extension, four reviews [21, 24–26] showed that telerehabilitation had a statistically significantly improved joint extension (P < 0.01), and two reviews [20, 30] showed that there was no statistically significant difference in joint extension between the two groups.

In terms of walking ability, six reviews [18–21, 23, 28] used the TUG test to evaluate the effect of telerehabilitation on walking ability. Three reviews [18, 21, 23] showed that telerehabilitation shortened the TUG test compared with standard rehabilitation, and two reviews [19, 28] showed no significant difference in TUG scores between the two groups. One review [20] conducted a descriptive analysis and reached no conclusions.

In terms of quality of life, three reviews [20, 28, 30] evaluated the effect of telerehabilitation on quality of life, and a meta-analysis of one medium-quality review [30] showed no significant difference in terms of changes in quality of life between the intervention and control groups.

### Evaluation results of secondary outcome indicators

#### Adverse events

Two reviews [20, 24] reported the influence of telerehabilitation on adverse events. Jansson et al. [20] showed that telerehabilitation had no significant effect on the occurrence of adverse events. Tsang et al. [24] reported that the rehospitalisation rate in an intervention group within 12 weeks was significantly lower than in a control group. However, the evidence quality was low.

#### Patient satisfaction

Four reviews [18, 19, 29, 30] evaluated the intervention effect of telerehabilitation on patient satisfaction. One review [18] showed that the telerehabilitation and control groups had similar patient satisfaction levels. Three reviews [19, 29, 30] showed that the changes in patient satisfaction were not statistically significant in either group.

#### Cost

Four reviews [18, 20, 24, 27] evaluated the effects of telerehabilitation on costs, and the results showed that telerehabilitation could reduce patient medical costs compared with expenses incurred in the control groups.

## Discussion

Telerehabilitation can provide patients with more flexible and convenient rehabilitation services using information technology, which compensates for shortcomings of conventional rehabilitation and is a complementary or alternative method to conventional nursing [9]. In this study, the AMSTAR-2, PRISMA, GRADE and ROBIS grades were used for the first time to review the effectiveness and safety of telerehabilitation in patients following TKA to provide more evidence for clinical decision making. Among the 13 systematic reviews and meta-analyses, 92.3% were published in the last 5 years, indicating increasing attention to telerehabilitation in recent years.

Based on the evaluation results of AMSTAR 2, the quality of the methodologies included in these reviews should be improved. The main issues identified in terms of critical weaknesses included item 2 (scheme registration in advance), item 4 (the comprehensive retrieval strategy) (n = 4, 30.8%), item 7 (the list of reasons for the exclusion of each document not being provided) (n = 13, 100%), item 13 (the risk of inclusion bias not considered when interpreting or discussing the findings) (n = 8, 61.5%) and item 15 (publication bias not being evaluated and discussed) (n = 7, 53.8%). Among the noncritical weaknesses, none of the systematic reviews described the source of funding for the included original research, and the study results may have been affected by the funding situation. Future systematic reviews and meta-analyses should strictly comply with the AMSTAR 2 standards to reduce methodological defects and bias and improve rigour.

According to the PRISMA 2020 assessment results, most reviews (76.9%) were relatively complete; however, none fully matched the quality of the PRISMA reports. The main issues were as follows: nine reviews (69.2%) did not have study protocol registration numbers, five (38.5%) did not provide other available materials and evidence certainty, four (30.8%) did not report study bias, and three (23.0%) did not perform certainty assessment. Future systematic reviews should follow the PRISMA report list to avoid these problems.

According to the ROBIS bias risk assessment results, domains 2 and 4 in the second stage had a high risk of bias. The main issues in determining and selecting research in domain 2 were as follows: most of the reviews were retrieved from the MEDLINE and Embase databases. However, conference reports and clinical trial registration platforms were not retrieved, and some reviews had restrictions on publication form and language, leading to bias. Shortcomings regarding data synthesis and result presentation in domain 4 included the inability to assess adherence to the predetermined scheme, the absence of sensitivity analysis and the potential unreliability of some results. In the third stage, all the reviews showed a high risk of bias, which may be related to the lack of a corresponding explanation and treatment of the partial bias risk in the second stage.

According to the GRADE evidence quality evaluation results, most of the 59 outcome indicators (55.9%) had low-quality or no high-quality evidence. The main factors affecting the evidence quality were the limitations of the original studies in terms of randomisation, blinding methods and allocation hiding. The second factor was publication bias, owing to the limited number of included studies or small sample sizes, lack of funnel plot analysis and other relevant considerations. In addition, due to the different intervention methods and measurement tools used in the included studies, heterogeneity was high, which affected the quality of evidence. A detailed description of the inclusion and exclusion criteria and appropriate subgroup analyses were required. In conclusion, clinical studies should focus on improving quality and standardising research methods to provide more reliable and objective data.

Our findings indicate that telerehabilitation positively affected walking ability, knee extension and costs for patients after TKA. Telerehabilitation was similar to conventional rehabilitation regarding the WOMAC score, quality of life and patient satisfaction. Telerehabilitation had no significant effect on pain or joint flexion. In addition, the effect of telerehabilitation on KOOS and adverse events in patients with TKA remains controversial. However, the above results should be treated with caution owing to the low quality of the studies assessed in systematic reviews and meta-analyses.

## Recommendations for future studies

Systematic reviews should follow the AMSTAR 2, PRISMA and ROBIS guidelines; protocol registration should be conducted before study initiation; research transparency should be enhanced; search strategies and databases should be comprehensive; a list should be provided when excluding the literature and reasons given; and the source of funding for the original study should be clarified. This study also found that the evidence quality of some original studies was not high. Therefore, sample sizes should be increased in future studies to reduce bias in randomisation, blinding methods and allocation hiding. Moreover, because telerehabilitation includes multiple types, a subgroup analysis should be performed when there is significant heterogeneity among the different types. Finally, the existing literature has not paid sufficient attention to patients’ adverse reactions or safety indicators after telerehabilitation. Future studies should also consider these indicators.

This study had some limitations. Owing to language limitations, this study only conducted searches in an English language databases; studies published in other languages may have been missed. There may have been duplications in the original studies included in the systematic reviews, which were not evaluated in this study. Telerehabilitation involves various intervention methods, and there was considerable heterogeneity among the different interventions. Only the descriptive analysis was used in this study, and no combined effect values were used for the quantitative analysis. Finally, although the AMSTAR-2, PRISMA 2020, ROBIS and GRADE systems were used for evaluation according to the appropriate standards, the evaluation process and results might remain subjective.

## Conclusion

In this study, telerehabilitation positively affected walking ability, knee extension and costs in patients who underwent TKA. Regarding patients’ quality of life, WOMAC scores and patient satisfaction, telerehabilitation had effects similar to conventional rehabilitation. However, these reported outcomes were influenced by the methodology, reporting quality, risk of bias and quality of evidence in the original studies. Therefore, the conclusions should be interpreted with caution. High-quality RCTs and systematic reviews are required to provide reliable evidence.

## Materials and methods

This study followed the Preferred Reporting Items for Systematic Reviews and Meta-Analyses (PRISMA) list [13] and was registered in the International Prospective Register of Systematic Reviews (PROSPERO) (registration number: CRD42023401152). The inclusion criteria were male or female patients aged ≥ 18 who underwent TKA.

The intervention groups comprised those who had undergone remote rehabilitation using the internet, smartphones, virtual reality or conference calls. The control groups received routine nursing and face-to-face rehabilitation therapy or were the blank control group. The primary outcome measures were pain, knee flexion and extension, timed up and go (TUG) test score, Western Ontario and McMaster Universities Osteoarthritis Index (WOMAC) score, knee injury and osteoarthritis outcome score (KOOS) and patient quality of life. The secondary outcome measures were adverse events, patient satisfaction and costs. The study type comprised systematic reviews of telerehabilitation in patients who underwent TKA with or without meta-analyses. The exclusion criteria comprised studies not published in English or Chinese, duplicate publications, conference abstracts, studies where the full text is unobtainable, studies mainly measuring the performance of applications and wearable devices and studies involving patients with other comorbidities in which TKA was not the main research topic.

### Retrieval databases

We searched PubMed, Cochrane Library, Embase, Cumulated Index to Nursing and Allied Health Literature, Web of Science, China National Knowledge Infrastructure and Veip databases from their establishment to 18 December 2022. A preliminary search was conducted to expand the search terms, and a combination of subject headings and free words was then used to search multiple databases. Finally, the PROSPERO website and references included in the literature were searched manually. The following search terms were used: knee replacement OR joint replacement, telemedicine OR remote rehabilitation OR internet OR video conference, video phone, virtual reality, WeChat, smartphone, application, wearable device, digital health, artificial intelligence, phone OR SMS and meta-analysis, systematic review, meta-analysis OR meta-analysis (see Additional file 1: Appendix Table 1 for specific search strategies).

### Literature screening and data extraction

#### Literature screening

After all the retrieved results were imported into EndNote X9 for duplication removal, two researchers independently screened the literature, first reading the title and abstract, excluding inconsistent literature, and then reading the full text to determine the suitability of the study for inclusion. The two researchers crosschecked and discussed any disagreements with a third person.

#### Data extraction

Two researchers separately extracted data from the literature that met the research criteria according to a preformulated Excel extraction table. The extracted data included the first author, year, country, number of original studies/sample size, study type, study subjects, intervention measures, control measures, outcome indicators, quality assessment tools and meta-analyses. In cases of disagreement, discussions were held with a third party.

### Evaluation of literature quality

#### Evaluation of methodological quality

Two authors used the A Measurement Tool to Assess Systematic Reviews 2 (AMSTAR 2) [14] scale to evaluate the methodological quality of the included studies (16 items in total). Each item was indicated by ‘yes’, ‘no’ and ‘partly yes’, and the key items were 2, 4, 7, 9, 11, 13 and 15. The final quality was divided into four grades as follows: (i) high quality, none or only one noncritical weakness; (ii) medium quality, more than one noncritical weakness; (iii) low quality, one critical flaw with or without noncritical weaknesses; and (iv) very low quality, more than one critical flaw with or without noncritical weaknesses. The results were checked after the evaluation, and any disagreement was resolved through discussion with a third person(fei wang).

#### Reporting quality evaluation

Two researchers evaluated each study’s quality using PRISMA [13], the preferred reporting method for determining items when assessing systematic reviews and meta-analyses. There were 27 items, including the title, abstract, introduction, methods, results, discussion and funding statements. The presence, partial presence or absence of each item was indicated by ‘yes’, ‘partially yes’ and ‘no’, with scores of 1, 0.5 and 0, respectively, with a total possible score of 27. A score of ≤ 15 was defined as a relatively severe information deficiency, 15–21 showed a certain reporting deficiency, and 21–27 indicated that the report was relatively complete. The results were checked after the evaluation, and discussions were held with a third person (fei wang)when the researchers disagreed.

#### Risk of bias assessment

The risk of bias was assessed by two researchers using the Risk of Bias in Systematic Reviews (ROBIS) scale [15], which consists of three stages. The first stage assessed the correlations (selected based on the situation). The second stage determined the risk of bias in the process of systematic review production in terms of four areas: the inclusion criteria, search and screening, data extraction and quality assessment and data synthesis and presentation of results, with a total of 21 items. The third stage determined the ROBIS as high risk, low risk or uncertain. Using ROBIS, we checked the results following this evaluation, and any disagreements were discussed with a third person(fei wang).

#### Grading of evidence quality

Two researchers used the standard Grading of Recommendations Assessment, Development and Evaluation (GRADE) system [16] to evaluate the risks of bias, imprecision, inconsistency, indirectness and publication bias. The evidence quality was divided into four grades: high, medium, low and very low [17]. After the evaluation, the results were checked, and any differences were discussed with a third person(fei wang).

### Data analysis methods

Descriptive methods were used because of the high heterogeneity of the included studies in terms of intervention methods and outcome indicators.

### Supplementary Information

**Additional file 1: Appendix Table 1.** Search strategy.

**Additional file 2: Appendix Table 2.** Exclude Articles.

## Data Availability

All data generated or analysed during this study are included in this article. Further enquiries can be directed to the corresponding author.
