# Supplementary material for: The efficacy and safety of telerehabilitation for patients following total knee arthroplasty: a overviews of systematic reviews
Source: Biomed Eng Online. 2023 Oct 8;22:97. doi: 10.1186/s12938-023-01158-z (PMC10560426; doi:10.1186/s12938-023-01158-z)
Supplement: Supplementary file 1 — Additional file 1: Appendix Table 1. Search strategy. [file 12938_2023_1158_MOESM1_ESM.docx]

**Appendix Table 1 Search strategy**

(1)PubMed

| **Search** | **Query** |
| --- | --- |
| #1 | (Arthroplasty, Replacement, Knee[Mesh] or Knee Prosthesis [Mesh] or Arthroplasty, Replacement or knee [Mesh]) OR (Arthroplasty, Knee Replacement[Title/Abstract] OR Knee Replacement Arthroplast*[Title/Abstract] OR Replacement Arthroplasties, Knee[Title/Abstract] OR Knee Arthroplasty, Total[Title/Abstract] OR Arthroplasty, Total Knee[Title/Abstract] OR Total Knee Arthroplasty[Title/Abstract] OR Replacement, Total Knee[Title/Abstract] OR Knee Replacement[Title/Abstract] |
| #2 | (Telemedicine [Mesh] or Telerehabilitation [Mesh] or Internet [Mesh] or Videoconferencing [Mesh] or Virtual Reality [Mesh] or Mobile Applications [Mesh] or Wearable Electronic Devices[Mesh] or Artificial Intelligence [mesh] or Text Messaging [mesh]) OR (Remote Rehabilitation*[Title/Abstract] OR Virtual Rehabilitation[Title/Abstract] OR Mobile Health[Title/Abstract] OR mHealth[Title/Abstract] OR Telehealth[Title/Abstract] OR eHealth[Title/Abstract] OR Video Conferenc*[Title/Abstract] OR video telephone[Title/Abstract] OR Reality, Virtual[Title/Abstract] OR Virtual Reality, Educational[Title/Abstract] OR WeChat[Title/Abstract] OR apps[Title/Abstract] OR applications[Title/Abstract] OR Mobile Apps[Title/Abstract] OR Smartphone[Title/Abstract] OR Wearable Devices[Title/Abstract] OR digital health[Title/Abstract] OR Intelligence, Artificial[Title/Abstract] OR telephone[Title/Abstract] OR social media[Title/Abstract] OR email[Title/Abstract]) |
| #3 | (((Meta-analysis[Title/Abstract] OR systematic review[Title/Abstract]) OR (review, systematic[MeSH Terms])) OR (meta analysis as topic[MeSH Terms])) OR (meta analy*[Title/Abstract] OR systematic review*[Title/Abstract]) Sort by: Publication Date |
| #4 | #1 and #2 and #3 |

（2）Web of Science

| **Search** | **Query** |
| --- | --- |
| #1 | TS=(Arthroplasty, Replacement, Knee or Knee Prosthesis or Arthroplasty or Arthroplasty, Replacement or knee) |
| #2 | AB=(Arthroplasty, Knee Replacement or Knee Replacement Arthroplast* or Replacement Arthroplasties, Knee or Knee Arthroplasty, Total Or Arthroplasty, Total Knee or Total Knee Arthroplasty or Replacement, Total Knee or Knee Replacement )) |
| #3 | #1 or #2 |
| #4 | TS=(Telemedicine or Telerehabilitation or Internet or Videoconferencing or Virtual Reality or Mobile Applications or Wearable Electronic Devices or Artificial Intelligence OR Text Messaging ) ) |
| #5 | AB=(Remote Rehabilitation* OR Virtual Rehabilitation OR Mobile Health or mHealth or Telehealth or eHealth or Video Conferenc* or video telephone or Reality, Virtual OR Virtual Reality, Educational or WeChat or apps OR applications or Mobile Apps or Smartphone or Wearable Devices or digital health or Intelligence, Artificial or telephone or social media or email )) |
| #6 | #4 or #5 |
| #7 | TS=(Meta-analysis OR systematic review or meta analy*or systematic review*) |
| #8 | #3 or #6 or #7 |

（3）Cochrane Library

| Search | Query |
| --- | --- |
| #1 | MeSH descriptor: [Arthroplasty, Replacement, Knee] explode all trees |
| #2 | MeSH descriptor: [Knee Prosthesis] explode all trees |
| #3 | MeSH descriptor: [Arthroplasty, Replacement or knee] explode all trees |
| #4 | (Arthroplasty, Knee Replacement or Knee Replacement Arthroplast* or Replacement Arthroplasties, Knee or Knee Arthroplasty, Total Or Arthroplasty, Total Knee or Total Knee Arthroplasty or Replacement, Total Knee or Knee Replacement):ti,ab,kw |
| #5 | #1 or #2 or #3or#4 |
| #6 | MeSH descriptor: [Telemedicine] explode all trees |
| #7 | MeSH descriptor: [Telerehabilitation] explode all trees |
| #8 | MeSH descriptor: [Internet] explode all trees |
| #9 | MeSH descriptor: [Videoconferencing] explode all trees |
| #10 | MeSH descriptor: [Virtual Reality] explode all trees |
| #11 | MeSH descriptor: [Mobile Applications] explode all trees |
| #12 | MeSH descriptor: [Wearable Electronic Devices] explode all trees |
| #13 | MeSH descriptor: [Artificial Intelligence] explode all trees |
| #14 | (Remote Rehabilitation* OR Virtual Rehabilitation OR Mobile Health or mHealth or Telehealth or eHealth or Video Conferenc* or video telephone or Reality, Virtual OR Virtual Reality, Educational or WeChat or apps OR applications or Mobile Apps or Smartphone or Wearable Devices or digital health or Intelligence, Artificial or telephone or social media or email):ti,ab,kw |
| #15 | #7 or #8 or #9 or #10 or #11 or #12 or #13 or #14 |
| #16 | MeSH descriptor: [Meta-Analysis] explode all trees |
| #17 | MeSH descriptor: [Systematic Review] explode all trees |
| #18 | (Meta analy*OR systematic review*):ti,ab,kw |
| #19 | #16 or#17 or #18 |
| #20 | #5 or #15 or #19 |

（4）Embase

| **Search** | **Query** |
| --- | --- |
| #1 | 'arthroplasty, replacement knee'/exp OR 'knee prosthesis'/exp OR 'arthroplasty'/exp OR 'arthroplasty,'/exp OR 'arthroplasty replacemen or knee '/exp |
| #2 | 'arthroplasty knee replacement' :ab,ti OR 'knee replacement arthroplast*':ab,ti OR' Replacement arthroplasties knee ' :ab,ti OR 'knee arthroplasty total ':ab,ti OR ' arthroplasty total knee ':ab,ti OR 'total knee arthroplasty ' :ab,ti OR 'replacement total knee':ab,ti OR 'knee replacement' :ab,ti |
| #3 | #1 or #2 |
| #4 | 'telemedicine ' :ab,ti OR ' telerehabilitation ':ab,ti OR ' internet ':ab,ti OR 'videoconferencing ':ab,ti OR 'virtual reality' :ab,ti OR 'mobile applications ' :ab,ti OR 'wearable electronic devices ' :ab,ti OR 'artificial intelligence ' :ab,ti OR 'text messaging ' :ab,ti |
| #5 | 'remote rehabilitation* ' :ab,ti OR ' virtual rehabilitation ' :ab,ti OR 'mobile health' :ab,ti OR mhealth ' :ab,ti OR 'telehealth ' :ab,ti OR 'ehealth ' :ab,ti OR ' video conferenc* ' :ab,ti OR 'video telephone ' :ab,ti OR ' reality virtual ' :ab,ti OR ' virtual reality educational ' :ab,ti OR ' wechat ' :ab,ti OR 'apps ' :ab,ti OR 'applications' :ab,ti OR 'mobile apps ' :ab,ti OR 'smartphone' :ab,ti OR ' wearable devices' :ab,ti OR 'digital health ' :ab,ti OR ' intelligence artificial' :ab,ti OR 'telephone' :ab,ti OR ' social media' :ab,ti OR 'email' :ab,ti |
| #6 | #4 or #5 |
| #7 | 'meta analysis' /exp |
| #8 | meta analysis (topic)' :ab,ti |
| #9 | 'systematic review'/exp |
| #10 | 'systematic review (topic)' :ab,ti |
| #11 | systematic AND review* :ab,ti |
| #12 | meta AND analy* :ab,ti |
| #13 | #7 or #8 #9 #10 or #11 or #12 |
| #14 | #3 and #6 and #13 |

（5）CINAHL：by EBSCO

| **Search** | **Query** |
| --- | --- |
| #1 | MH=(Arthroplasty, Replacement, Knee or Knee Prosthesis or Arthroplasty or Arthroplasty, Replacement or knee) OR (Arthroplasty, Knee Replacement or Knee Replacement Arthroplast* or Replacement Arthroplasties, Knee or Knee Arthroplasty, Total Or Arthroplasty, Total Knee or Total Knee Arthroplasty or Replacement, Total Knee or Knee Replacement)) |
| #2 | MH=Telemedicine or Telerehabilitation or Internet or Videoconferencing or Virtual Reality or Mobile Applications or Wearable Electronic Devices or Artificial Intelligence OR Text Messaging (Remote Rehabilitation* OR Virtual Rehabilitation OR Mobile Health or mHealth or Telehealth or eHealth or Video Conferenc* or video telephone or Reality, Virtual OR Virtual Reality, Educational or WeChat or apps OR applications or Mobile Apps or Smartphone or Wearable Devices or digital health or Intelligence, Artificial or telephone or social media or email ) |
| #3 | MH=(Meta-analysis OR systematic review or meta analy*or systematic review*) |
| #4 | #1 AND #2 AND #3 |

（6）Sinomed

| **Search** | **Query** |
| --- | --- |
| #1 | "Knee replacement "[Common field: intelligent] OR" Joint replacement "[common field: intelligent] |
| #2 | "Telemedicine "[commonly used field: intelligent] OR" remote rehabilitation "[commonly used field: intelligent] OR "Internet "[commonly used field: intelligent] OR" video conferencing "[commonly used field: intelligent] OR "video phone "[commonly used field: intelligent] OR" virtual reality "[commonly used field: intelligent] OR "Wechat "[common field: intelligent] OR" smart phone "[common field: intelligent] OR "application "[common field: intelligent] |
| #3 | "Wearable device "[common field: intelligent] OR "digital health "[common field: intelligent] OR" artificial intelligence "[common field: intelligent] OR "phone "[common field: intelligent] OR" SMS "[common field: intelligent] |
| #4 | #2 or #3 |
| #5 | "Systematic Review "[commonly used field: intelligent] OR" meta-analysis "[commonly used field: intelligent] OR "meta-analysis" or "meta-analysis "[commonly used field: intelligent] |
| #6 | #1 and #4 and #5 |

(7)CNKI

Subject: knee replacement or joint replacement results Retrieved in: Subject: Telemedicine or remote rehabilitation or Internet or video conferencing or video phone or virtual reality or wechat or smartphone or app or wearable device or sensor or digital health or artificial intelligence or phone or SMS

(8)VIP

Title or keywords: (knee replacement or joint replacement) and title or keywords: (Telemedicine or tele-rehabilitation or Internet or video conference or video call or virtual reality or wechat or smart phone or app or wearable device or digital health or artificial intelligence or phone or SMS) and title or keywords: (Systematic review or meta-analysis or meta-analysis or meta-analysis)
