# Supplementary material for: The efficacy and safety of telerehabilitation for patients following total knee arthroplasty: a overviews of systematic reviews
Source: Biomed Eng Online. 2023 Oct 8;22:97. doi: 10.1186/s12938-023-01158-z (PMC10560426; doi:10.1186/s12938-023-01158-z)
Supplement: Supplementary file 2 — Additional file 2: Appendix Table 2. Exclude Articles. [file 12938_2023_1158_MOESM2_ESM.docx]

**Appendix Table 2 Exclude Articles**

| **First Author, year** | **Title** | **Reasons for exclusion** |
| --- | --- | --- |
| Agostini M,2015 | Telerehabilitation and recovery of motor function: a systematic review and meta-analysis | Not only TKA patients. |
| Aïm F，2015 | Effectiveness of Virtual Reality Training in Orthopaedic Surgery | Not only TKA patients. |
| Berton A，2020 | Virtual Reality, Augmented Reality, Gamification, and Telerehabilitation: Psychological Impact on Orthopedic Patients' Rehabilitation | Not an SR |
| Constantinescu D，2022 | The role of commercially available smartphone apps and wearable devices in monitoring patients after total knee arthroplasty: a systematic review. | Interventions not based on telerehabilitation |
| Gumaa M，2019 | Is Virtual Reality Effective in Orthopedic Rehabilitation? A Systematic Review and Meta-Analysis | Not only TKA patients. |
| Follis S，2020 | Comparison of wearable sensor to traditional methods in functional outcome measures: A systematic review | Not only TKA patients. |
| Petersen W，2021 | A systematic review about telemedicine in orthopedics | Not only TKA patients. |
| Bahadori S，2018 | Smartphone apps for total hip replacement and total knee replacement surgery patients: a systematic review. | Interventions not based on telerehabilitation |
| Velayati F，2020 | A Systematic Review of the Effectiveness of Telerehabilitation Interventions for Therapeutic Purposes in the Elderly | TKA patients was not the main research object |
| Bäcker HC，2022 | A Review of Functional Outcomes after the App-Based Rehabilitation of Patients with TKA and THA | TKA patients was not the main research object |
| Wang X,2018 | Digital disruptive technology for rehabilitation following elective surgery for low back pain, knee and hip osteoarthritis: A systematic review and meta-analysis | [conference summary](javascript:;) |
| Velayati F，2020 | A Systematic Review of the Effectiveness of Telerehabilitation Interventions for Therapeutic Purposes in the Elderly. | TKA patients was not the main research object |
| Chaudhry H，2021 | How Satisfied Are Patients and Surgeons with Telemedicine in Orthopaedic Care during the COVID-19 Pandemic? A Systematic Review and Meta-Analysis | TKA patients was not the main research object |
| Dávila Castrodad IM，2019 | Rehabilitation protocols following total knee arthroplasty: a review of study designs and outcome measures | Interventions not based on telerehabilitation |
| Amin J，2022 | Rehabilitation Professional and Patient Satisfaction with Telerehabilitation of Musculoskeletal Disorders: A Systematic Review | TKA patients was not the main research object |
| Panda S，2015 | Telerehabilitation and total knee arthroplasty: A systematic review and meta-analysis of randomised controlled trials | [conference summary](javascript:;) |
| Pastora-Bernal JM，2017 | Evidence of Benefit of Telerehabitation After Orthopedic Surgery: A Systematic Review | TKA patients was not the main research object |
| Fahey E，2021 | Telemedicine in Orthopedic Surgery: A Systematic Review of Current Evidence | TKA patients was not the main research object |
| Brigo E，2022 | Using Telehealth to Guarantee the Continuity of Rehabilitation during the COVID-19 Pandemic: A Systematic Review | TKA patients was not the main research object |
